# Estudio del patrón lesional de los traumas graves en Navarra (2010-2019)

**DOI:** 10.23938/ASSN.1085

**Published:** 2024-08-28

**Authors:** Eider Arbizu Fernández, Arkaitz Galbete, Tomás Belzunegui Otano, Mariano Fortún Moral, Alfredo Echarri Sucunza

**Affiliations:** 1 Servicio Navarro de Salud-Osasunbidea Hospital Universitario de Navarra Servicio de Urgencias Generales Pamplona España; 2 Navarrabiomed Grupo de investigación en Pacientes Politraumatizados Pamplona España; 3 Instituto de Investigación Sanitaria de Navarra (IdisNa) Pamplona España; 4 Universidad Pública de Navarra (UPNA) Departamento de Estadística, Informática y Matemáticas Institute of Smart Cities Pamplona España; 5 Servicio Navarro de Salud-Osasunbidea Gerencia de Atención Primaria Subdirección de Urgencias de Navarra y Dirección Técnica de la Atención a la Urgencia Vital Navarra España

**Keywords:** Registros, Traumatismo, Puntaje de Gravedad del Traumatismo, Mortalidad, Sexo, Records, Wounds and injuries, Injury severity scale, Mortality, Sex

## Abstract

**Fundamento:**

El objetivo de este estudio es describir los traumas graves (TG) en Navarra y analizar sus diferencias por mortalidad, sexo y mecanismo lesional.

**Material y métodos:**

Estudio transversal de TG (gravedad ≥3) registrados en Navarra desde 2010 a 2019. Se analizó el tipo de TG, su intencionalidad, mecanismo y región anatómica afectada. Se calculó el riesgo (OR) de TG según distintas variables.

**Resultados:**

Se incluyeron 2.609 pacientes con TG, con media de edad 54,7 años (0-101) y 70,9 % varones. Predominaron los TG contusos (94,7 %) y accidentales (84 %) causados por caídas (46,5 %) y accidentes de coche (18,4 %). Las mujeres sufrieron más caídas y atropellos y los hombres más accidentes de moto, bicicleta, arma blanca/de fuego y contusiones. La mayoría de TG se registraron en cabeza y tórax. Las lesiones en cabeza fueron significativamente más frecuentes en fallecidos y en mujeres, y las lesiones en tórax en personas fallecidas *in situ* y en hombres. Las causas más frecuentes de TG en cabeza fueron caídas de baja altura y armas de fuego y, en torax, los accidentes de coche y las caídas de altura. El riesgo de TG disminuyó con la edad y se multiplicó por 2-3 en pacientes fallecidos.

**Conclusión:**

Se han identificado diferencias por sexo en intencionalidad, tipo de traumatismo y mecanismo del TG. Globalmente, las lesiones en cabeza y tórax son más letales, y las abdominales y de extremidades/anillo pélvico se observaron en muertes tempranas, sugiriendo una afectación tan extensa y grave que dificulta su tratamiento y manejo.

## Abstract (en)

**Background:**

The aim of this study is to describe major trauma cases in Navarre and analyze differences based on morta-lity groups, sex, and mode of injury.

**Methods:**

Cross-sectional study of major traumas (severity ≥3) registered in Navarre between 2010 and 2019. We analyzed the type of trauma, intentionality, the mode of injury, and the affected anatomical area. The *odds ratio* for major trauma associated with different variables was calculated.

**Results:**

The study included 2,609 patients; mean age was 54.7 years (0-101) and 70.9% were male. A predominance of accidental (84%) / blunt (94.7%) major traumas was recorded, primarily resulting from falls (46.5%) and car accidents (18.4%). Women experienced more falls and pedestrian accidents, while men had more motorcycle, bicycle, knife/firearm accidents, and contusions. Most major traumas affected the head and thorax. Head trauma was significantly more common in deceased individuals and women, while thoracic trauma was more frequent in patients who died on-site and in men. Head injuries were caused by falls from low heights and firearms, whereas thoracic injuries resulted from car accidents and falls from height. The risk of major trauma decreased with age; deceased patients were between two and three times more likely to present major traumas in all anatomical areas.

**Conclusions:**

Gender differences are observed in intentionality, type, and mode of injury. Head and thoracic injuries are potentially life-threatening and abdominal and extremity/pelvic ring injuries are associated with early deaths. This suggests that the extent and severity of these injuries complicate treatment and management.

## INTRODUCCIÓN

El trauma grave (TG) es el conjunto de lesiones provocadas simultáneamente por una energía externa, dando lugar a un cuadro clínico que afecta a diferentes órganos, aparatos o sistemas, mostrando una gravedad progresiva y comprometiendo seriamente las funciones vitales^1^; su definición varía según las fuentes.

El TG es una pandemia mundial^2^^,^^3^ y una de las principales causas de discapacidad y muerte, tanto es así que representa el 18% de la carga mundial de enfermedad^3^. Las causas, los tipos lesionales, la gravedad y su pronóstico varían, y los factores de riesgo dependen directamente de las características sociales, culturales y/o económicas^4^.

Las muertes por TG ocurren tanto a nivel hospitalario (MH) como extrahospitalario (MEH). En España, se produce una MH por cada cinco MEH y en Europa la tasa desciende hasta 1:9^5^. A pesar de ser las más frecuentes, la bibliografía sobre MEH es escasa.

La escala AIS (*Abbreviated Injury Scale*) es un sistema de codificación de gravedad global de lesiones consensuado que clasifica cada lesión según la importancia relativa de la región del cuerpo, en una escala ordinal de 6 puntos^6^^,^^7^. Es la más utilizada en el mundo desde su creación en 1971 por la AAAM (*Association for the Advancement of Automotive Medicine*) y su uso es fruto de la corriente emprendida hace ya años para la unificación de criterios de recogida de datos a nivel mundial^6^. A lo largo de los años, los datos codificados según la AIS han sido introducidos en la mayoría de los registros de trauma existentes, creando una fuente indispensable de información para uso científico^8^. La AIS es la base científica de la escala de gravedad de las lesiones basada en la anatomía corporal utilizada en el Registro *Major Trauma* de Navarra (RMTN), que ha sido comparado con otros registros demostrando su validez^9^.

El TG es de comunicación obligatoria por decreto de la Comisión Europea^10^ de 2016, y se define como la identificación de lesiones significativas en tres o más puntos en dos o más regiones anatómicas AIS diferentes, junto con una o más variables adicionales de los cinco parámetros fisiológicos^11^. Por lo tanto, el estudio de la gravedad de estas lesiones proporciona la oportunidad de definir las lesiones anatómicas concretas que generan las muertes en pacientes con TG, convirtiéndose su estudio en necesidad^12^^,^^13^.

El objetivo de este estudio es catalogar las lesiones anatómicas registradas mediante una escala de gravedad preestablecida y derivada de la AIS, en los pacientes con TG registrados en el RMTN desde 2010 hasta 2019, analizando sus diferencias por mortalidad, sexo y mecanismo lesional.

## MATERIAL Y METODOS

Estudio transversal retrospectivo realizado en los servicios de Urgencias y en el Instituto de Medicina Legal y Forense de la comunidad foral de Navarra (España) con datos de pacientes con TG recogidos entre el 1 de enero de 2010 y el 31 de diciembre de 2019.

Según datos del INE (Instituto Nacional de Estadística), Navarra cuenta con un área de 10.391 Km^2^, con una población de 633.023 habitantes al inicio del estudio y 649.946 al final del mismo^14^. Los servicios de urgencias situados en la capital, Pamplona, están compuestos por un hospital terciario como centro de referencia para la atención del TG, el Centro de Coordinación SOS Navarra (donde se gestionan los recursos de emergencia a través de la activación mediante el teléfono 112), tres ambulancias dotadas de médico a bordo y un helicóptero sanitario. Cuenta además con dos hospitales comarcales (en Tudela y en Estella), cada uno con un recurso de ambulancia medicalizada. El resto del sistema de emergencias lo componen ambulancias de soporte vital básico dotadas de personal técnico de emergencias sanitarias, y servicios de urgencias rurales (SUR) compuestos por dos personas especialistas una en Medicina y otra en Enfermería.

Se incluyeron pacientes lesionados por agentes externos de cualquier intencionalidad que fueron atendidos por los diferentes recursos de urgencias, con lesiones que puntuaron >15 puntos^15^^-^^20^ en la escala NISS (*New Injury Severity Score*), escala que selecciona las tres lesiones más graves de cada región anatómica. Este fue el punto de corte establecido para TG debido a que se observó que aumentaba la sensibilidad -sin disminuir la especificidad- respecto a la definición anterior (>15 puntos en la *Injury Severity Score*, ISS) establecida por Boyd y col en 1987^21^. Los criterios de exclusión fueron: pacientes admitidos tras más de 24 horas, casos de asfixia e inmersión, ahorcamientos, envenenamientos, intoxicaciones y quemados sin otras lesiones traumáticas^21^.

Los pacientes que cumplían con los criterios de selección se registraron en el RMTN^22^^-^^23^, base de datos activa desde 2010 y alimentada por todos los profesionales médicos intervinientes en el manejo del TG, por el personal médico forense del Instituto de Medicina Legal de Navarra en el caso de pacientes que fallecen *in situ* y/o en las primeras horas, y por el personal médico de los servicios de urgencias hospitalarios y extrahospitalarios para el resto de los pacientes.

Los datos se introducen de forma manual en la aplicación del RMTN, a la que el personal médico previamente autorizado accede mediante usuario y contraseña, abierta en un navegador convencional en equipos informáticos conectados a la red institucional del Gobierno de Navarra. El registro lo realiza el personal prehospitalario cuando identifica un posible caso de TG, introduciendo -mediante los despegables disponibles en la aplicación- datos personales, fecha, centro receptor e información prehospitalaria: puntuaciones *Revised Trauma Score* (RTS) y *Glasgow Coma Score* (GCS), mecanismo e intención de la lesión. A continuación, el personal hospitalario diagnostica al paciente y completa el registro con las puntuaciones ISS, NISS, RTS y comorbilidad previa. Los fallecimientos son registrados por el médico forense, calculando el ISS/NISS a partir de la autopsia. Este proceso está supervisado constantemente por el administrador, quien vela por el cumplimiento de los criterios de exclusión e inclusión y contrasta las personas ingresadas en los hospitales de la comunidad foral y las registradas en el RMTN, con el fin de evitar la no inclusión de pacientes^23^.

Antes de iniciarse con el uso del RMTN, hubo una formación previa para conocer los criterios de estilo Utstein, modelo estandarizado de recogida unificada de variables que hace comparables las bases de datos para posibilitar investigaciones futuras^21^^,^^24^. El personal que alimenta el RMTN tiene experiencia en el manejo de este tipo de pacientes y, en los puntos donde había dudas, se llegó a un consenso para el registro.

Las variables recogidas fueron:

- Demográficas: edad (años), sexo (hombre, mujer);
- Traumatismo: tipo (contuso, penetrante), intencionalidad (accidental, autoinfligida, agresión, otros) y mecanismo (coche o autobús o camión, moto, bicicleta, atropello, arma de fuego, arma blanca, contusión con objetos, caída desde altura propia, caída desde altura >3 metros, otros).
- Gravedad: el RMTN utiliza una escala elaborada por el equipo investigador a partir de la escala AIS (Anexo I) que mide la gravedad anatómica de 148 lesiones una vez identificados los pacientes objetivo, por lo que no influyen los cambios en los parámetros fisiológicos secundarios a las lesiones y al tratamiento de este tipo de pacientes^1^. Divide el cuerpo en nueve regiones (cabeza, cara, cuello, tórax, abdomen, columna vertebral, extremidad superior, extremidad inferior y externo de otro tipo (abrasiones, quemaduras o laceraciones) y asigna un valor de 1 a 6 a partir del estado de gravedad del paciente (1 = menor, 2 = moderado, 3 = severo sin compromiso vital, 4 = severo con compromiso vital, 5 = crítico, 6 = incompatible con la vida)^25^. Para este estudio las regiones se agruparon en seis (cabeza/cuello, cara, tórax, abdomen, extremidades/anillo pélvico y superficie corporal) porque en algunos grupos las lesiones son muy poco frecuentes.

Para documentar la variable dependiente, mortalidad (fallecimiento, supervivencia), tras el evento traumático se realizó el seguimiento del paciente mediante la historia clínica informatizada del Gobierno de Navarra.

El protocolo se desarrolló de acuerdo con las normas éticas y fue aprobado por el Comité Ético de Investigación Clínica y con medicamentos de Navarra (PI_2020/60). No se requirió consentimiento informado.

Las características de los pacientes se describieron utilizando la media y la desviación estándar (DE) para variables cuantitativas y frecuencia y porcentaje para variables categóricas. Las comparaciones entre grupos se llevaron a cabo mediante las pruebas t de Student para variables continuas y Chi-cuadrado (**χ^2^**) o prueba exacta de Fisher para variables categóricas, según correspondiera. Se ajustó un modelo logístico multivariable para presencia de lesión en la escala de gravedad ≥3 en cada región, excluyendo superficie corporal por falta de casos para poder ajustar el modelo, incluyendo como variables predictoras edad, sexo, mortalidad y mecanismo lesional. Todos los contrastes fueron bilaterales con un nivel de significación de 0,05; el software utilizado para realizar estos análisis fue IBM SPSS Statistics v28.0.

## RESULTADOS

Durante los 10 años del estudio se incluyeron 2.609 pacientes con trauma grave (NISS >15 y AIS modificada ≥3 en cada región anatómica).

La media de edad fue 54,7 años (rango 0 a 101) y el 70,9% fueron hombres. La mayoría sufrió un traumatismo contuso (94,7%) de forma accidental (84%), y entre las causas predominaron las caídas (46,5%) y el coche (18,4%) (Tabla 1).

Se observaron algunas diferencias significativas por sexo: los hombres fueron una media de 12 años más jóvenes, con doble frecuencia de traumatismos penetrantes y de agresiones. Globalmente, los mecanismos lesionales difirieron, ya que las mujeres sufrieron el doble de caídas de propia altura y 1,6 veces más atropellos, mientras que los hombres sufrieron cuatro veces más traumatismos por moto, ocho por bicicleta, tres por arma blanca o de fuego y siete veces más contusiones (Tabla 1).

**Tabla 1:** Edad de los pacientes con trauma grave y características de los mismos, global y por sexo

Variable	Total	Sexo		p (χ^2^)
		Mujer	Hombre	
	n=2.609	n=759 (29,1%)	n=1.850 (70,9%)	
Edad, media (DE)	54,7 (23,7)	63,3 (24,9)	51,2 (22,3)	<0,001^*^
*Traumatismo, n (%)*				
*Tipo*				<0,001
Penetrante	139 (5,3)	19 (2,5)	120 (6,5)	
Contuso	2.470 (94,7)	740 (97,5)	1.730 (93,5)	
*Intencionalidad*				0,012
Accidental	2.245 (84)	660 (87,0)	1.585 (85,7)	
Autoinfligido	291 (11,2)	89 (11,7)	202 (10,9)	
Agresión/Otros	73 (2,2)	10 (1,4)	63 (3,4)	
*Mecanismo*				<0,001
Coche	479 (18,4)	137 (18,1)	342 (18,5)	
Moto	179 (6,9)	16 (2,1)	163 (8,8)	
Bicicleta	141 (5,4)	14 (1,8)	127 (6,9)	
Atropello	176 (6,7)	70 (9,2)	106 (5,7)	
Arma de fuego	59 (2,3)	6 (0,8)	53 (2,3)	
Arma blanca	42 (1,6)	5 (0,7)	37 (2,0)	
Contusión	151 (5,8)	8 (1,1)	143 (7,7)	
Caída altura propia	774 (29,7)	351 (46,2)	423 (22,9)	
Caída >3 m altura	438 (16,8)	125 (16,5)	313 (16,9)	
Otros	170 (6,5)	27 (3,5)	143 (7,7)	

*: test t de Student; DE: desviación estándar.

**Figura 1:**
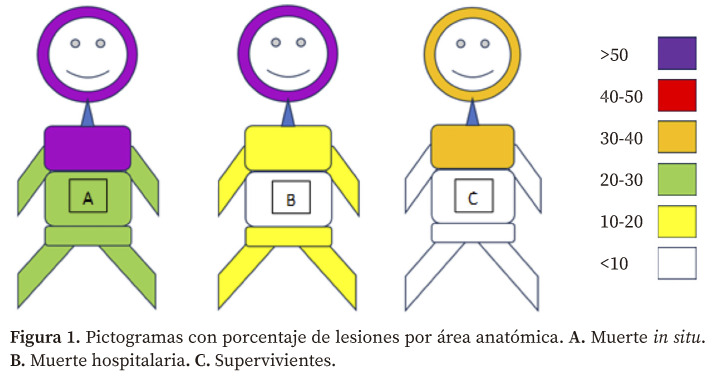
Pictogramas con porcentaje de lesiones por área anatómica. **A.** Muerte *in situ*. **B.** Muerte hospitalaria**. C.** Supervivientes.

Las personas que fallecieron mostraban >50% de lesiones con puntuación de gravedad ≥3 en la cabeza frente al 30-40% mostrado por los supervivientes. Las personas fallecidas *in situ* también mostraban una mayor frecuencia de lesiones en tórax (Fig. 1, Tabla 2).

Globalmente, la cabeza y el tórax son las regiones con mayor proporción de lesiones de gravedad ≥3. Hubo diferencias significativas (p<0,001) por sexo en relación a los traumas graves en cabeza (un 8,3% más frecuentes en mujeres) y en tórax (un 6,6% más frecuentes en hombres). En la Tabla 2 se describen dichas lesiones por mortalidad y sexo.

**Tabla 2:** Frecuencia de lesiones de gravedad ≥3 por regiones anatómicas, global y por categorías de mortalidad

Región	Global	Mortalidad [n (%)]			p (χ^2^)
		*In situ*	Hospital	Supervivientes	
*Mujeres*					
Cabeza	350 (46,8)	74 (52,5)	112 (62,9)	164 (38,2)	<0,001
Cara	15 (2,0)	5 (3,5)	5 (2,8)	5 (1,2)	0,111^*^
Tórax	217 (29,0)	76 (53,9)	28 (15,7)	113 (26,3)	<0,001
Abdomen	98 (13,1)	45 (31,9)	12 (6,7)	41 (9,6)	<0,001
EE/Anillo pélvico	101 (13,5)	44 (31,2)	16 (9,0)	41 (9,6)	<0,001
Superficie corporal	3 (0,4)	3 (2,1)	0 (0,0)	0 (0,0)	0,007^*^
*Hombres*					
Cabeza	708 (38,5)	237 (49,4)	134 (55,4)	337 (30,2)	<0,001
Cara	41 (2,2)	19 (4,0)	2 (0,8)	20 (1,8)	0,008
Tórax	654 (35,6)	239 (49,8)	55 (22,7)	360 (32,3)	<0,001
Abdomen	253 (13,8)	122 (25,4)	19 (7,9)	112 (10,0)	<0,001
EE/Anillo pélvico	222 (12,1)	99 (20,6)	27 (11,2)	96 (8,6)	<0,001
Superficie corporal	8 (0,4)	7 (1,5)	0 (0,0)	1 (0,1)	0,001^*^

*: test exacto de Fisher; EE: extremidades.

También se observaron diferencias en el perfil de las regiones anatómicas afectadas dependiendo del mecanismo de lesión. La presencia de lesiones graves en cabeza fue más prevalente en lesiones por arma de fuego y caídas de baja altura tanto en mujeres como en hombres, y menos en lesiones por arma blanca. En el caso de las mujeres, las lesiones en la cabeza y en el tórax en los accidentes de moto se igualan con un 31,3% al igual que en los atropellos con un 34,3%. El tórax se vio afectado en accidentes de coche y caídas desde altura, en su mayoría en ambos sexos, y específicamente en hombres en accidentes de moto (54%) (Tabla 3).

**Tabla 3:** Frecuencia [n (%)] de lesiones con gravedad ≥3 según regiones anatómicas, por mecanismo y por sexo

Sexo	Región	Coche	Moto	Bicicleta	Atropello	Arma de fuego
*Mujeres*	Cabeza	45 (33,1)	5 (31,3)	6 (42,9)	24 (34,3)	4 (66,7)
	Cara	3 (2,2)	0	0	0 (0,0)	1 (16,7)
	Tórax	78 (57,4)	5 (31,3)	5 (37,3)	24 (34,3)	0
	Abdomen	32 (23,5)	1 (6,3)	1 (7,1)	10 (14,3)	0 (0,0)
	EE/Anillo pélvico	25 (18,4)	4 (25,0)	1 (7,1)	18 (25,7)	1 (16,7)
	Externo	2 (1,5)	0	0	0 (0,0)	0 (0,0)
	*Región*	*Arma blanca*	*Contusión*	*Caída baja*	*Caída >3 m*	*Otros*
	Cabeza	0	2 (28,6)	207 (60,0)	47 (37,9)	10 (40,0)
	Cara	0	0	5 (1,4)	5 (4,0)	1 (4,0)
	Tórax	0	3 (42,9)	31 (9,0)	61 (49,2)	10 (40,0)
	Abdomen	0	1 (14,3)	7 (2,0)	42 (33,9)	4 (16,0)
	EE/Anillo pélvico	1 (20,0)	2 (28,6)	7 (2,0)	37 (29,8)	5 (20,0)
	Externo	0	0	0	0	1 (4,0)
*Sexo*	*Región*	*Coche*	*Moto*	*Bicicleta*	*Atropello*	*Arma de fuego*
*Hombres*	Cabeza	100 (29,3)	39 (23,9)	40 (31,5)	47 (44,2)	25 (47,2)
	Cara	7 (2,1)	3 (1,8)	6 (4,7)	4 (3,8)	6 (11,3)
	Tórax	181 (53,1)	88 (54,0)	49 (38,6)	43 (40,6)	12 (22,6)
	Abdomen	73 (21,4)	23 (14,1)	13 (10,2)	19 (17,9)	7 (13,2)
	EE/Anillo pélvico	78 (22,9)	24 (14,7)	7 (5,5)	18 (17,0)	0
	Externo	3 (0,9)	1 (0,6)	0	0	0
	*Región*	*Arma blanca*	*Contusión*	*Caída baja*	*Caída >3 m*	*Otros*
	Cabeza	5 (13,9)	56 (39,2)	230 (55,3)	123 (39,5)	43 (30,3)
	Cara	0	3 (2,1)	1 (0,2)	9 (2,9)	2 (1,4)
	Tórax	10 (27,8)	44 (30,8)	40 (9,6)	134 (43,1)	53 (37,3)
	Abdomen	8 (22,2)	20 (14,0)	11 (2,6)	57 (18,3)	22 (15,5)
	EE/Anillo pélvico	4 (11,1)	11 (7,7)	7 (1,7)	54 (17,4)	19 (13,4)
	Externo	0	0	1 (0,2)	0	3 (2,1)

EE: extremidades.

En la Tabla 4 se ajusta un modelo de regresión logística para el riesgo de presencia de lesión con gravedad ≥3 en las diferentes regiones anatómicas. Se observa que a mayor edad hay significativamente menor riesgo de lesión grave, mientras que por sexo se encontraron diferencias únicamente en extremidades y pelvis, donde los hombres mostraron menor riesgo de lesión grave. Al analizar los grupos de mortalidad, se constató que los pacientes que fallecieron *in situ* presentaron un riesgo de lesiones en todas las áreas anatómicas entre doble y triple que aquellos que sobrevivieron. Por contra, los pacientes que fallecieron en el hospital solo mostraron diferencias significativas en relación con las lesiones en la cabeza y extremidades/pelvis respecto al mecanismo de la lesión, se observó que las caídas de altura propia y las contusiones se relacionaron con mayor presencia de lesiones graves en la cabeza, mientras que los diferentes accidentes de tráfico (coche y moto) mostraron mayor riesgo de lesión grave en tórax.

**Tabla 4:** Modelos logísticos multivariables para presencia de lesión con puntuación ≥3 en la escala de gravedad para las diferentes regiones anatómicas

Variables	OR (IC95%)				
	Cabeza	Cara	Tórax	Abdomen	Extremidades/pelvis
*Edad*	1,00	0,99	1,00	0,99	1,00
	(0,99-1,00)	(0,97-1,00)	(1,00-1,01)	(0,98-0,998)	(0,99-1,00)
*Sexo*					
Mujer	1,00	1,00	1,00	1,00	1,00
Hombre	0,9	0,8	0,98	0,78	0,69
	(0,74-1,09)	(0,43-1,51)	(0,79-1,21)	(0,59-1,03)	(0,52-0,91)
*Mortalidad*					
Supervivientes	1,00	1,00	1,00	1,00	1,00
*In situ*	3,09	2	1,94	2,73	2,44
	(2,49-3,84)	(1,05-3,81)	(1,57-2,40)	(2,10-3,55)	(1,86-3,21)
Hospital	2,39	1,6	0,83	1,18	1,78
	(1,88-3,05)	(0,65-3,92)	(0,62-1,11)	(0,77-1,82)	(1,19-2,65)
*Mecanismo*					
Coche	1,00	1,00	1,00	1,00	1,00
Moto	0,93	0,92	1,04	0,68	0,85
	(0,62-1,40)	(0,25-3,46)	(0,74-1,49)	(0,41-1,12)	(0,53-1,37)
Bicicleta	1,66	2,75	0,63	0,57	0,31
	(1,09-2,52)	(0,94-8,03)	(0,43-0,94)	(0,31-1,05)	(0,15-0,66)
Atropello	1,65	1,24	0,55	0,84	0,94
	(1,13-2,40)	(0,38-4,08)	(0,38-0,80)	(0,52-1,34)	(0,60-1,46)
Arma de fuego	1,44	5,2	0,15	0,32	0,05
	(0,82-2,54)	(1,82-14,8)	(0,08-0,30)	(0,14-0,75)	(0,01-0,33)
Arma blanca	0,27	-	0,25	0,78	0,47
	(0,10-0,71)		(0,12-0,53)	(0,34-1,77)	(0,18-1,24)
Contusión	1,7	1,09	0,41	0,68	0,4
	(1,14-2,53)	(0,29-4,07)	(0,27-0,60)	(0,40-1,14)	(0,22-0,75)
Caída altura propia	4,1	0,57	0,1	0,14	0,08
	(3,08-5,47)	(0,18-1,76)	(0,07-0,14)	(0,08-0,25)	(0,04-0,14)
Caída >3 m	1,48	1,65	0,67	1,1	0,94
	(1,12-1,97)	(0,72-3,78)	(0,51-0,88)	(0,80-1,51)	(0,68-1,30)
Otros	1,01	0,87	0,46	0,62	0,59
	(0,68-1,50)	(0,24-3,22)	(0,32-0,67)	(0,38-1,01)	(0,36-0,97)

## DISCUSIÓN

En este estudio se incluyen todas las lesiones catalogadas de gravedad ≥3 registradas en el RMTN sobre un total de 2.609 pacientes de la comunidad foral de Navarra durante 10 años (2010-2019). Está basado en el primer registro de datos poblacional puesto en marcha en torno a los pacientes politraumatizados en España, cuyo amplio número de datos recogidos en estos 10 años puede aportar información precisa sobre la evolución de los casos. Además, cuenta con los datos de personas fallecidas *in situ*, datos que no constan en otros estudios a pesar de su importancia, como reflejan Alberto Hernández-Tejedor y col^26^.

Las regiones anatómicas más afectadas fueron la cabeza y el tórax, mostrando una afectación similar en fallecidos *in situ* y un aumento considerable en la cabeza en el grupo hospitalario, para luego igualarse en los supervivientes. Sin embargo, no se observa una afectación considerable en el abdomen en ninguno de los casos, al igual que en las extremidades, y la afectación de la cara es anecdótica. En relación con estos resultados, y en concordancia con el estudio de Perry Li^27^, parece razonable priorizar la exploración torácica, ya sea física o ecográfica, sobre la abdominal, en la atención clínica más temprana por parte de los servicios extrahospitalarios. Actualmente, la exploración pulmonar ecográfica está en último puesto en el protocolo E-Fast *(Extended Focused Assesment with Sonography in Trauma*)^28^^,^^29^.

Las mujeres presentan una mayor afectación de la cabeza, similar a los resultados de Hernández-Tejedor y col^26^, mientras que los hombres muestran una mayor afectación del tórax y tienen menos riesgo de tener una lesión grave en pelvis y extremidades, pudiendo estar relacionadas con diferencias hormonales^26^. No se observaron grandes diferencias en el resto de las regiones, y tampoco en la mortalidad global, tal y como mencionaron Schoeneberg y col^30^. Pero sí las hay en intencionalidad y tipo de traumatismo, siendo más frecuentes las agresiones y traumatismos penetrantes en hombres y los autoinfligidos y traumatismos contusos en mujeres, pudiendo tener relación con la diferencia de conductas según género, aunque no se puede concluir por falta de estudios al respecto. Sin embargo, también podrían relacionarse con el mecanismo lesional, ya que las caídas de propia altura fueron más frecuentes en mujeres y los accidentes de tráfico de diversas causas en hombres^26^^,^^31^. Las caídas de propia altura en mujeres causan el doble de traumatismos graves que en los hombres, pudiendo relacionarse con la mayor esperanza de vida femenina y el aumento de fragilidad en pacientes por encima de los 60 años^32^, pero se precisan más estudios para corroborarlo.

En relación con los mecanismos lesionales y las regiones afectadas, se observa que la cabeza tiene un riego mayor de afectación en el caso de la bicicleta y la moto, por lo que el uso regulado por ley del casco en ambos casos (en España, el uso obligatorio se implementó en 1992 para las motos y en 2021 para las bicicletas) debiera ser revisado para analizar si ha supuesto algún cambio desde su aplicación. Sin embargo, en nuestra base de datos no hay registro del uso del casco, a pesar del elevado uso poblacional registrado^33^. En la mitad de los casos de lesiones por arma de fuego resulta afectada la cabeza, proporción similar a la descrita por Taylor y col^34^. El tórax es la región más afectada en los accidentes de coche y moto, mientras que el abdomen, la pelvis y las extremidades son las más afectadas en accidentes de coche y en las caídas de altura.

Entre las limitaciones del estudio está el hecho de no utilizar la escala de gravedad AIS original, debido a la imposibilidad de contar con codificadores y la dificultad de acceso a dicha escala. Una limitación de la escala utilizada es que las lesiones de diferentes regiones, puntuadas igual, no necesariamente tienen la misma gravedad ni aumentan proporcionalmente a la puntuación de gravedad asignada a la lesión. Sin embargo, la escala utilizada califica la gravedad de igual manera que la escala AIS, proporcionando resultados muy similares, como se ha demostrado en diversos estudios^5^^,^^12^^,^^13^^,^^22^. Igualmente, sigue siendo un reto automatizar el volcado de datos a la aplicación del RMTN desde la historia clínica informatizada del Servicio Navarro de Salud-Osasunbidea. Otra limitación es que algunos casos de trauma grave pueden no ser registrados, por escapar a la búsqueda realizada^33^ o por atenderse en centros privados, de los que carecemos de datos y que, debido a la organización sanitaria, atienden un número poco significativo en comparación con la muestra analizada.

En conclusión, de forma global se han identificado como posibles lesiones letales aquellas de gravedad ≥3 situadas en cabeza y tórax. Además, las muertes más tempranas ocurren en presencia de lesiones abdominales y de extremidades/anillo pélvico, lo que puede indicar que la afectación es tan extensa y grave que, una vez establecidas, su tratamiento y manejo son difíciles. Se observaron diferencias por sexo en cuanto a intencionalidad, tipo de traumatismo y algunos mecanismos de lesión. Por tanto, este estudio realizado sobre lesiones graves puede ayudar a mejorar las medidas preventivas mediante el desarrollo de protocolos de acción específicos y dirigidos ya que, a diferencia de las personas fallecidas *in situ*, el manejo de pacientes de los otros grupos podría beneficiarse del conocimiento previo de las lesiones graves más frecuentemente generadas.

## Data Availability

Datos no disponibles.

## ANEXO 1 Escala de gravedad elaborada por el equipo investigador a partir de la escala AIS (*Abbreviated Injury Scale*)

**Table:**

Localización de las lesiones		Grado
*Cabeza*		
1	Cefalea/mareos secundarios al traumatismo craneoencefálico	1
2	Rigidez cervical sin fractura ni luxación	1
3	Laceración cuero cabelludo	1
4	Amnesia del accidente	2
5	Letargia, estupor, obnubilación. Puede ser despertado con estímulo verbal	2
6	Inconsciencia <1 hora	2
7	Fractura simple de bóveda	2
8	Contusión tiroidea	2
9	Lesión de plexo braquial	2
10	Dislocación o fractura de la apófisis espinosa o transversa de vértebras cervicales	2
11	Compresión menor/ fractura (<20%) de vértebra cervical	2
12	Fractura de hioides	2
13	Inconsciencia 1-6 horas	3
14	Inconsciencia <1 hora con déficit neurológico	3
15	Fractura de base craneal	3
16	Fractura de bóveda conminuta o hundimiento	3
17	Contusión cerebral/ hemorragia subaracnoidea	3
18	Laceración de íntima o trombosis de la arteria carótida	3
19	Contusión laríngea o faríngea	3
20	Dislocación o fractura de cuerpo laminar, pedículo o faceta de vértebra cervical	3
21	Aplastamiento de >1 vértebra o 20% de altura anterior	3
22	Inconsciencia 1-6 horas con déficit neurológico	4
23	Inconsciencia 6-24 horas (lesión axonal difusa leve)	4
24	Respuesta adecuada solo al estímulo doloroso	4
25	Fractura craneal con hundimiento >2 cm, desgarro de dura o pérdida de masa	4
26	Hematoma intracraneal subdural o epidural <50 mL	4
27	Lesión incompleta de médula cervical	4
28	Aplastamiento laríngeo	4
29	Laceración/trombosis de arteria carótida con déficit neurológico	4
30	Fractura de base craneal en bisagra	4
31	Inconsciencia con movimientos inapropiados	5
32	Inconsciencia >24 horas (lesión axonal difusa moderada o grave)	5
33	Lesión de tronco	5
34	Hematoma intracraneal subdural o epidural >50 mL	5
35	Lesión completa de médula cervical de C4 o más baja	5
36	Rotura de cartílagos tiroides y/o cricoides	5
37	Fractura aplastamiento	6
38	Aplastamiento/laceración de tronco cerebral	6
39	Decapitación	6
40	Aplastamiento de médula, laceración o transección total, con o sin fractura de C3 o superior	6
*Cara*
41	Abrasión corneal	1
42	Laceración de lengua	1
43	Fractura nasal o de rama mandibular	1
44	Fractura dental, avulsión o dislocación	1
45	Fractura cigomática, orbitaria, cuerpo mandibular o subcondilar	2
46	Fractura tipo Lefort I	2
47	Laceración de esclera o córnea	2
48	Laceración de nervio óptico	3
49	Fractura tipo Lefort II	3
51	Fractura tipo Lefort III	4
*Tórax*		
52	Fractura costal	1
53	Rigidez columna dorsal	1
54	Contusión de caja torácica	1
55	Contusión esternal	1
56	Fractura de 2-3 costillas	2
57	Fractura esternal	2
58	Dislocación o fractura de espinosa o proceso transversal de espina dorsal	2
59	Aplastamiento menor, fractura de <20% vertebral dorsal	2
60	Contusión o laceración pulmonar (1 lóbulo)	3
61	Hemo- o neumotórax unilateral (<1.000 mL)	3
62	Rotura de diafragma. Fractura >3 costillas	3
63	Lesión de íntima, laceración menor o trombosis de arteria innominada o subclavia	3
64	Quemadura menor por inhalación	3
65	Dislocación o fractura de cuerpo laminar, pedículo o faceta de vértebra dorsal	3
66	Fractura aplastamiento de >1 vértebra dorsal o más del 20 % de la altura	3
67	Contusión de médula con signos neurológicos transitorios	3
68	Volet costal <5 costillas	3
69	Contusión o laceración pulmonar multilobar	4
70	Hemo/neumomediastino. Hemo/neumotórax bilateral	4
71	Volet costal >5 costillas. Contusión miocárdica	4
72	Neumotórax a tensión. Hemotórax >1.000 mL	4
73	Fractura traqueal. Desgarro de la íntima (aorta)	4
74	Laceración mayor de arteria subclavia o innominada	4
75	Síndrome medular incompleto	4
76	Hemo- o neumotórax unilateral (>1.000 mL)	4
77	Laceración menor aorta (intima)	4
78	Rotura diafragmática	4
79	Laceración mayor de aorta (con hemorragia confinada a mediastino)	5
80	Laceración cardiaca	5
81	Rotura bronquial o de tráquea	5
82	Volet costal bilateral/ quemaduras por inhalación, requiriendo ventilación mecánica	5
83	Separación laringo-traqueal	5
84	Laceración pulmonar multilobar con hemotórax a tensión o hemotórax >1.000 mL	5
85	Laceración o lesión medulares completa	5
86	Ruptura auricular	5
87	Laceración mayor de aorta (con hemorragia no confinada a mediastino)	6
88	Aplastamiento masivo de tórax	6
89	Ruptura ventricular	6
*Abdomen*		
90	Abrasión/contusión superficial/laceración de escroto, vagina, vulva, periné	1
91	Rigidez de espina lumbar	1
92	Hematuria	1
93	Contusión superficial/laceración estómago, mesenterio, vejiga, uréter, uretra.	2
94	Contusión menor, laceración de riñón, hígado, bazo, páncreas.	2
95	Contusión de duodeno, colon.	2
96	Dislocación o fractura de espinosa o proceso transverso de vértebra lumbar	2
97	Fractura aplastamiento menor (<20%) vértebra lumbar	2
98	Lesión de raíz de nervio	2
99	Laceración superficial duodeno, colon, recto	3
100	Perforación intestino delgado, mesenterio, vejiga, uréter, uretra	3
101	Contusión mayor o laceración menor con implicación de vasos mayores o >1.000 mL de riñón, hígado, bazo, páncreas	3
102	Laceración menor de arteria o vena ilíaca	3
103	Hematoma retroperitoneal	3
104	Dislocación o fractura de cuerpo laminar, faceta o pedículo de vértebra lumbar	3
105	Fractura aplastamiento >1 vértebra o >20% de su altura anterior	3
106	Contusión medular con signos neurológicos transitorios	3
107	Perforación de estómago, duodeno, colon, recto	4
108	Perforación con pérdida de tejido estómago, vejiga, intestino delgado, uréter	4
109	Laceración hepática mayor	4
110	Laceración mayor de arteria o vena ilíaca	4
111	Síndrome medular incompleto	4
112	*Abruption* placenta	4
113	Laceración mayor con pérdida de tejido o gran contaminación de duodeno, colon, recto	5
114	Rotura compleja hepática, bazo, riñón, páncreas	5
115	Lesión medular completa	5
116	Transección de torso	6
*Extremidades/anillo pélvico*		
117	Contusión de codo, hombro, rodilla, tobillo	1
118	Fractura/luxación de dedos	1
119	Esguinces	1
120	Fractura de húmero, radio, cúbito, rótula, tibia, clavícula, escápula, carpo, metarcarpo, calcáneo, tarso, metatarso, rama púbica o fractura pélvica	2
121	Luxación de codo, hombro, mano...	2
122	Laceración mayor de músculo, tendones	2
123	Laceración menor/desgarro de íntima de arteria axilar, braquial, poplítea	2
124	Laceración menor/desgarro de íntima en vena axilar, poplítea, femoral	2
125	Fractura pélvica conminuta	3
126	Fractura abierta de húmero, radio, cúbito o tibia	3
127	Fractura de fémur	3
128	Luxación de rodilla, tobillo, cadera, codo. Amputación por debajo rodilla o en extremidades superiores	3
129	Rotura ligamentos rodilla. Laceración nervio ciático	3
130	Laceración menor/desgarro de íntima de arteria femoral	3
131	Laceración mayor/trombosis arteria axilar o poplítea, vena axilar, poplítea o femoral	3
132	Amputación antebrazo	3
133	Fractura aplastamiento pélvico	4
134	Amputación/Síndrome de aplastamiento traumático por encima de rodilla	4
135	Laceración mayor de arteria femoral o braquial	4
136	Amputación brazo	4
137	Fractura aplastamiento pélvica abierta	5
*Externo*		
138	Abrasión/contusiones <26 cm en cara/manos, <51 cm en cuerpo	1
139	Laceraciones superficiales <6 cm en cara/manos <11 cm en cuerpo	1
140	Quemadura de primer grado	1
141	Quemaduras de 2º o 3º grado <10%	1
142	Abrasión/contusiones <25 cm en cara/manos, >50% en cuerpo	2
143	Laceración >5 cm en cara/manos, >10 cm en cuerpo	2
144	Quemaduras de 2º o 3º grado del 10-19% de superficie corporal	2
145	Quemaduras de 2º o 3º grado del 20-29% de superficie corporal	3
146	Quemaduras de 2º o 3º grado del 30-39% de superficie corporal	4
147	Quemaduras de 2º o 3º grado del 40-89% de superficie corporal	5
148	Quemaduras de 2º o 3º grado de más del 89% de superficie corporal	6
